# Rapid Carbonation for Calcite from a Solid-Liquid-Gas System with an Imidazolium-Based Ionic Liquid

**DOI:** 10.3390/ijms150711350

**Published:** 2014-06-25

**Authors:** Abdul-Rauf Ibrahim, Jean Bosco Vuningoma, Yan Huang, Hongtao Wang, Jun Li

**Affiliations:** Department of Chemical and Biochemical Engineering, College of Chemistry and Chemical Engineering, National Engineering Laboratory for Green Chemical Productions of Alcohols, Ethers and Esters, Xiamen University, Xiamen 361005, China; E-Mails: ghrauf@gmail.com (A.-R.I.); v29johannes@gmail.com (J.B.V.); yanh1236@gmail.com (Y.H.); wanght@xmu.edu.cn (H.W.)

**Keywords:** calcite, carbon dioxide, carbonation, solid-liquid-gas, ionic liquid

## Abstract

Aqueous carbonation of Ca(OH)_2_ is a complex process that produces calcite with scalenohedral calcite phases and characterized by inadequate carbonate species for effective carbonation due to the poor dissolution of CO_2_ in water. Consequently, we report a solid-liquid-gas carbonation system with an ionic liquid (IL), 1-butyl-3-methylimidazolium bromide, in view of enhancing the reaction of CO_2_ with Ca(OH)_2_. The use of the IL increased the solubility of CO_2_ in the aqueous environment and enhanced the transport of the reactive species (Ca^2+^ and CO_3_^2−^) and products. The presence of the IL also avoided the formation of the CaCO_3_ protective and passivation layer and ensured high carbonation yields, as well as the production of stoichiometric rhombohedral calcite phases in a short time.

## 1. Introduction

Calcium carbonate (CaCO_3_) is an inorganic chemical that occurs in different crystalline polymorphs at ambient pressure. These are anhydrous phases of vaterite, calcite and aragonite and hydrated phases of monohydrocalcite and hexahydrocalcite (ikaite) [1]. Anhydrous CaCO_3_ is generally classified as rhombic calcite, needle-like aragonite or spherical vaterite, among which calcite is the most stable phase under normal atmospheric conditions [2]. The formation of any of the polymorphs is strictly guided by the temperature, supersaturation and pH of reaction solution [3]. However, chameleonic phase transformation was found between calcite, aragonite and vaterite, normal to abnormal and *vice versa*, in a CO_2_ expanded ethanol-water solution system applied for the solid-liquid-gas carbonation (SLGC) of Ca(OH)_2_ [4]. CaCO_3_ is used for several industrial applications and by living organisms for bone development [5]. For instance, it is used as an extender and to meliorate stability and exposure to resistance in paints [6,7]. As such, global consumption and demand for CaCO_3_ continues to increase [8,9]. Industrially, calcite is usually produced by the SLGC route, where CO_2_ is bubbled through a Ca(OH)_2_ slurry or slake lime [10,11,12,13]. Although the mechanism for the precipitation of CaCO_3_ from SLGC is debated, many investigators have shown that the absorption of CO_2_ is the rate determining step in the SLGC with a Ca(OH)_2_ suspension [10,11]. Current synthesis procedures are slow, energy intensive and less efficient, leading to low conversion yields. Several methods for synthesizing CaCO_3_ effectively and with various properties using additives and surfactants [14,15], block copolymers [16,17] and acids [18,19] have been explored.

The excellent role of ionic liquids (ILs) as reaction media and catalysts is common knowledge, among which, imidazolium-based ILs are the most researched group. Studies have shown that the solubility of CO_2_ in this group of ILs increases with increased pressure [20,21]. For example, Sudha and Khanna [22] in a molecular modeling of the solubility of CO_2_ in several ILs reported the solubility of CO_2_ in 1-butyl-3-methylimidazolium bromide, Bmim[Br], to be a 0.31 molar fraction. Once the absorption of CO_2_ is a rate determining step in the SLGC with the Ca(OH)_2_ suspension and CO_2_ is very soluble in ILs, coupling an SLGC system with an IL will enhance the absorption of CO_2_ and lead to higher conversion yields.

Previously, we conducted systematic research on a solid-gas carbonation (SGC) involving a dry Ca(OH)_2_ system with a solid ionic liquid (SIL) and high-pressure/supercritical CO_2_ and achieved the complete conversion of Ca(OH)_2_ to CaCO_3_ in 5 min [23,24]. The SIL served as the catalyst carrier and facilitated the effective dissolution of CO_2_, leading to the syntheses of stoichiometric rhombohedral calcite phases [23,24]. In the current study, we report an SLGC involving a Ca(OH)_2_ slurry system using an imidazolium-based IL in view of enhancing carbonation efficiency. The study searches for rapid carbonation for the production of nano-CaCO_3_ and the capture of CO_2_, since CO_2_ capture is an important industrial application [23]. The IL was used in the current work as a reaction media to increase the solubility of CO_2_ and to enhance the kinetics of the reaction in the liquid phase. Note that the IL is very miscible with water and can easily be recovered.

## 2. Results and Discussion

Precipitation of CaCO_3_ from aqueous carbonation of Ca(OH)_2_ is an exothermic and complex process with an overall reaction equation as [5,7]:

Ca(OH)_2(*aq*)_ + CO_2(*aq*)_ → CaCO_3(*s*)_ ↓ + H_2_O_(*l/v*)_ (1)

Traditionally, SLGC produces calcite with a scalenohedral lattice {2 1 −1} [25,26] and is characterized by excess calcium species (Ca^2+^) as a result of the limited solubility of CO_2_ in water [27,28,29], leading to inadequate carbonate species (CO_3_^2−^) necessary for producing the stoichiometric rhombohedral phase [24,27]. Thus, any process that enhances the solubility of CO_2_ and the dissolution of Ca(OH)_2_ can positively affect the reaction rate and result in higher conversion yields. Therefore, increasing the solubility of CO_2_ using an IL to facilitate the effective dissolution of Ca(OH)_2_ will not only enhance carbonation yields, but also facilitate and produce the stoichiometric rhombohedral calcite phase [24,27].

### 2.1. Effect of Temperature

**Figure 1:**
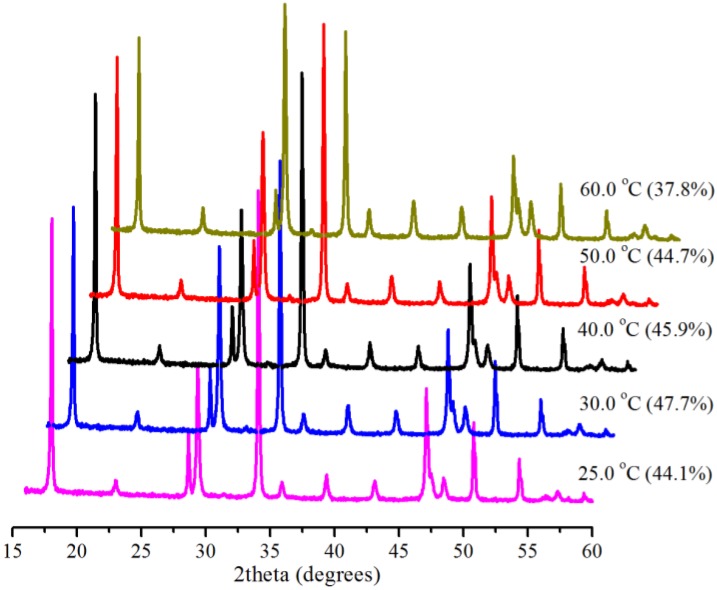
XRD (X-ray diffractometry) patterns and corresponding conversions (in brackets) for samples produced with 1-butyl-3-methylimidazolium bromide (Bmim[Br]) at various temperatures with 0.1 MPa in 5 min.

The effect of temperature on SLGC is well-known [7], and lower temperature is commonly adopted to increase the solubility of CO_2_ in water. We investigated the effect of temperature (25.0, 30.0, 40.0, 50.0 and 60.0 °C) at atmospheric pressure (0.1 MPa) in 5 min with 0.5 g Bmim[Br]/5.0 g Ca(OH)_2_ and 10 mL water. X-ray diffractometry (XRD) patterns of the produced samples and the corresponding conversions of Ca(OH)_2_ at the different temperatures are shown in Figure 1. As can be seen, the conversion increased from 44.1% at 25.0 °C to only 45.9% at 40.0 °C and then decreased slightly to 37.8% at 60.0 °C, revealing that temperature did not have an obvious effect on the system and that the IL took effect. The highest conversion (47.7%) for the 0.1 MPa reactions, although not complete, is about the best value reported for identical systems with shorter reaction times in the literature, indicating the availability of the IL.

**Figure 2:**
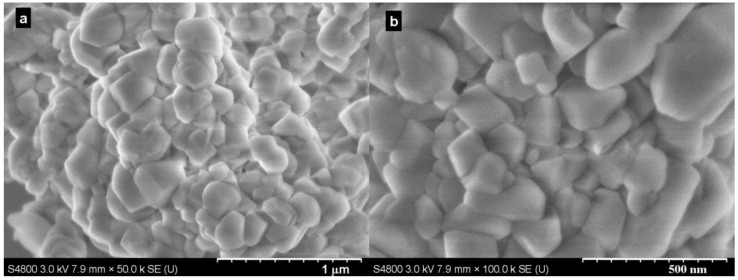
SEM (scanning electron microscope) images of samples produced at (**a**) 40.0 °C and (**b**) 60.0 °C with 0.1 MPa in 5 min.

The samples were also characterized by scanning electron microscope (SEM) to investigate their morphology (typically shown in Figure 2). The SEM images revealed that irregular rhombohedral calcite (ICCD-PDF-2: 01-086-2334) particles were produced at the conditions investigated. Additionally, there was no unique and distinct difference between the particles produced.

### 2.2. Effect of Pressure

**Figure 3:**
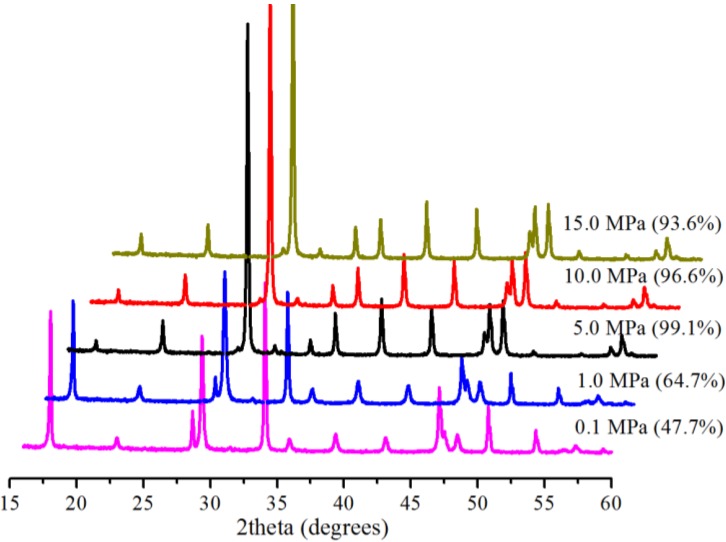
XRD patterns and corresponding conversions (in brackets) for samples produced with Bmim[Br] at various pressures at 30.0 °C in 5 min.

Figure 3 shows the effect of pressure on the conversion at 30.0 °C in 5 min with 0.5 g Bmim[Br]/5.0 g Ca(OH)_2_ and 10 mL water. At 0.1 MPa, there was incomplete conversion, because a 47.7% conversion was realized. This was evidenced by the presence of the characteristic peaks for both the starting material (at 18.0° and 34.2°) and the product (at 29.4°). A similar phenomenon was witnessed for the reaction at 1.0 MPa, resulting in a 64.7% conversion. However, at 5.0 MPa, the major peaks of the starting material almost disappeared, leading to pronounced major peaks of the product and substantial conversion (99.1%). Yet, a further increase of the pressure reduced the conversion slightly (Figure 3). The presence of the Bmim[Br] increased the conversions at 0.1 and 1.0 MPa (Figure 3). The quantity of dissolved CO_2_ needed to produce CO_3_^2−^ species for carbonation is restricted by the low solubility of CO_2_ in water at low pressures [5], but the Bmim[Br] enhanced the availability of the CO_3_^2−^ species, due to the high solubility of CO_2_ in the IL [22] and led to the high conversion yields.

**Figure 4:**
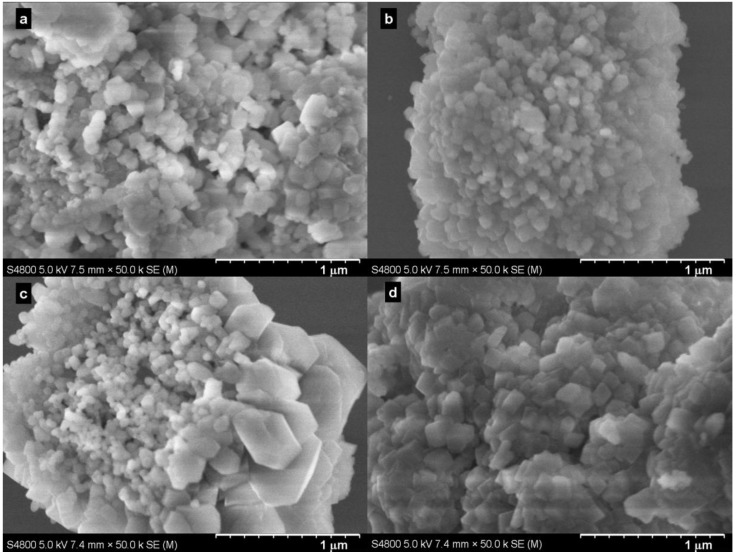
SEM images of samples produced with Bmim[Br] at (**a**) 0.1 MPa; (**b**) 1.0 MPa; (**c**) 5.0 MPa and without Bmim[Br] at (**d**) 5.0 MPa in 5 min at 30.0 °C.

Figure 4 shows typical SEM images of the samples produced at various pressures. The images confirmed the rhombohedral, but agglomerated, nature of the particles produced. A comparison of the particles produced at 5.0 MPa with Bmim[Br] (Figure 4c) and without Bmim[Br] (Figure 4d) revealed that the former sample consisted of the majority of less agglomerated and irregular, but smaller (≈60 nm) particles with a minority of bigger (≈300 nm) rhombohedral ones, while the latter sample had almost uniform, but highly agglomerated and bigger (≈100 nm) rhombohedral particles. Note that the conversion without the IL at 5.0 MPa and 30.0 °C in 5 min was 52.7%.

### 2.3. Effect of Reaction Time

**Figure 5:**
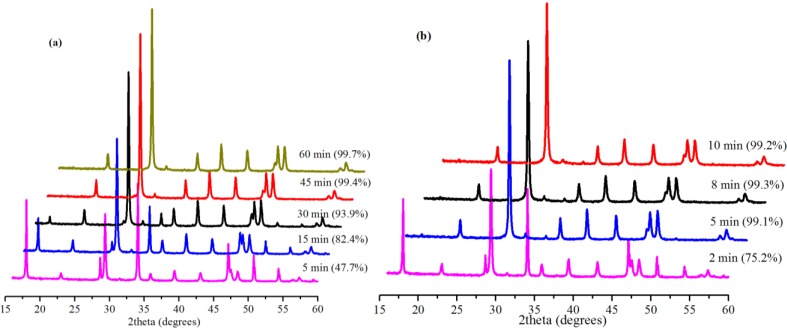
XRD patterns and corresponding conversions (in brackets) for samples produced with Bmim[Br] with different reaction times at (**a**) 0.1 MPa and (**b**) 5.0 MPa.

The extent of carbonation reaction is a function of time; hence, the effect of reaction time on the rate of conversion was investigated. Figure 5 shows the effect of reaction time on the conversion of Ca(OH)_2_ with 0.5 g Bmim[Br]/5.0 g Ca(OH)_2_ and 10 mL water at 30.0 °C for 0.1 and 5.0 MPa. At 0.1 MPa, the slow reaction led to low conversion (26.3%) after 5 min without Bmim[Br]. The conversion increased to 34.6% in 30 min and 65.3% in 60 min without the IL. However, it was 47.7% in 5 min when the IL was introduced, but increased to 93.9% in 30 min and then 99.7% after 60 min. Obviously, the effect of the IL on the conversion at this low pressure was significant especially, within 30 min. The conversion increased from 34.6% in 30 min without the IL to 93.9% (≈59.3% increment) when the IL was added.

Among other parameters, increasing the working pressure of CO_2_ increases its solubility and, by extension, the availability of CO_3_^2−^ species in the SLGC for better yield [5]. In this regard, several experiments were carried out at 5.0 MPa with the Bmim[Br] to investigate the effect of time. For these cases, the reactions were very fast (almost complete conversions) within 5 min. Even so, when the reaction was monitored over a very short time (2 min), the positive influence of the IL was evident. The reaction resulted in a 75.2% conversion demonstrating the ability of the IL to facilitate faster reaction in the initial stages of the carbonation at medium pressure (5.0 MPa). Furthermore, a 47.7% conversion was obtained at 0.1 MPa in 5 min, whereas the conversion was 99.3% at 5.0 MPa, confirming the fast nature of the reaction in the presence of the IL at the medium pressure.

**Figure 6:**
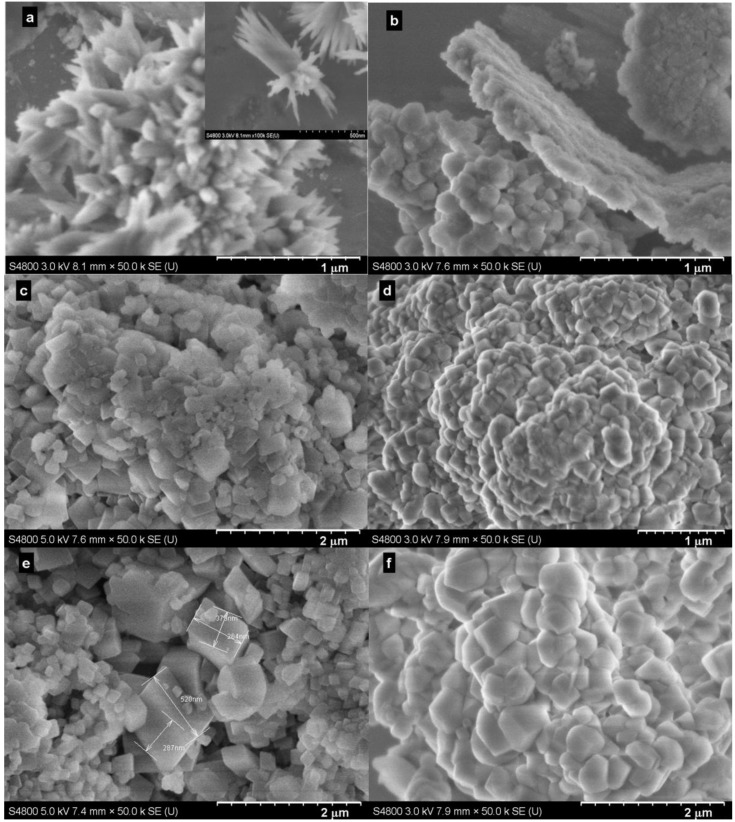
SEM images of the samples produced at 30.0 °C and 0.1 MPa. (**a**) with Bmim[Br] and (**b**) without Bmim[Br] in 5 min; (**c**) with Bmim[Br] and (**d**) without Bmim[Br] in 30 min; (**e**) with Bmim[Br] and (**f**) without Bmim[Br] in 60 min.

Representative SEM images for the samples produced at selected times are shown in Figure 6. Generally, and as expected, the samples produced with the Bmim[Br] exhibited well-defined particles (Figure 6a,c,e) compared to those produced without the IL (Figure 6b,d,f). Additionally, the effect of time on morphology was clearly visible. For instance, whereas the sample produced with the IL in 5 min consisted of a mixture of flower-like calcite (≈50 nm) and bundle-like hexagonal (inset) Ca(OH)_2_ particles (≈50 × 900 nm; Figure 6a), those produced in 30 min showed a perfect mixture of smaller (≈50 nm) and larger (≈400 nm) rhombohedral calcites (Figure 6c). However, the SEM images (Figure 6) indicated the obvious increase of particle size following the reaction time. Note that Figure 4a and Figure 6a are different images of the same sample; Figure 6a was deliberately taken to show both Ca(OH)_2_ and calcite particles, whereas Figure 4a showed only calcite particles.

### 2.4. Effect of Amount of Water

**Figure 7:**
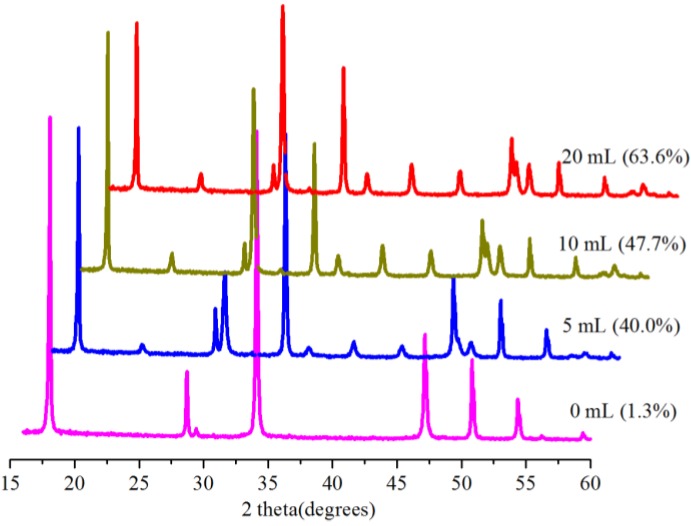
XRD patterns and corresponding conversions (in brackets) for samples produced with the Bmim[Br] and various amounts of water at 0.1 MPa and 30.0 °C in 5 min.

Water is very vital for carbonation reactions, and the role of water has been reported accordingly [5,23]. The influence of the amount of water on conversion was thus investigated at 0.1 MPa and 30.0 °C in 5 min with 0.5 g Bmim[Br]/5.0 g Ca(OH)_2_. For the zero-milliliter (0 mL) water, the IL was dried under vacuum for 36 h to remove traces of adsorbed water, and no additional water was added into the reactor during the reaction; so it was a dry carbonation (SGC) system [23]. The conversion results are shown in Figure 7, revealing increased conversion with an increasing amount of water. A conversion of 40.0% was obtained using 5.0 mL as opposed to 1.3% for the reaction without water (0 mL), but the conversion increased to 63.6% with 20.0 mL of water. Note that these were slurry systems and, as such, suspensions; hence the mentioning of the presence of water instead of concentrations or solutions.

### 2.5. Effect of Amount of Bmim[Br]

**Figure 8:**
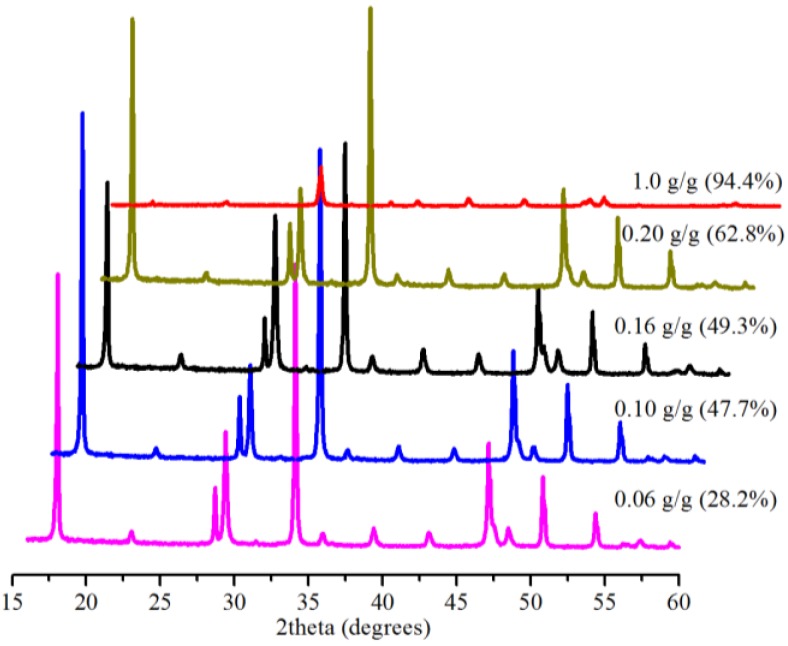
XRD patterns and corresponding conversions (in brackets) for samples produced with 10 mL water and various amounts of Bmim[Br] in 5 min.

The presence of Bmim[Br] was shown to have a significant effect on the conversion at the low (0.1 MPa) and medium (5.0 MPa) pressures in 5 min (see Section 2.2 and Section 2.3). Therefore, we investigated the effect of the mass ratio of the IL to Ca(OH)_2_ on the conversion at 0.1 MPa within 5 min at 30.0 °C. Figure 8 shows the conversion results of the samples produced, revealing increased conversion from 28.2% at 0.06 g IL/g Ca(OH)_2_ to 94.4% at 1.0 g IL/g Ca(OH)_2_. We are mindful of the comparatively expensive nature of ILs. However, about a 30% conversion of Ca(OH)_2_ at 0.1 MPa with just 6% (0.06 g IL/1 g Ca(OH)_2_) of the IL and a 99.1% conversion at 5.0 MPa with 10% (0.5 g IL/5 g Ca(OH)_2_) of the IL in 5 min, perhaps, justify the use of the IL in terms of cost.

### 2.6. Significance of Bmim[Br] and the Mechanism

Enhanced dissolution of CO_2_ is among the key parameters that control carbonation reactions, and for SLGC, enhancing the solubility of CO_2_ in water is particularly important. As such, the use of the IL was expected to enhance the production of the CO_3_^2−^ from the effective dissolution of CO_2_ in the IL [22] and affect conversion yields. The influence of the IL on conversion was evident at lower pressure. For example, with 0.1 MPa at 30.0 °C in 5 min, the reaction without the IL gave a conversion of 26.3% against about 50% with the IL (Figure 8). Furthermore, the effect of the IL on the particle size and degree of agglomeration at the medium pressure (5.0 MPa) was noticeable. The sample produced with the IL was a mixture of smaller (≈60 nm) and bigger (≈300 nm) rhombohedral particles with less agglomeration (Figure 4c), whereas the particles synthesized without the IL were bigger rhombohedrals (≈100 nm) with more agglomeration (Figure 4f). The IL also had a positive influence on the conversion (99.1%) in 5 min at the medium pressure, however; its availability was prominent when the reaction was monitored for 2 min (75.0% conversion), indicating that it induced a faster reaction within the initial stage of the carbonation. On the other hand, reaction with 0.10 g IL/g Ca(OH)_2_ in 5 min resulted in a conversion of 47.7%. A decreased amount of the IL (0.06 g IL/g Ca(OH)_2_ in 5 min), as expected, only worsened the conversion yield (28.2%), while an increased amount of the IL (1.0 g IL/g Ca(OH)_2_ in 5 min) gave a conversion (94.4%) greater than 90.0%. Considering the cost implications of ILs, 1.0 g IL/g Ca(OH)_2_ in 5 min for a 94.4% conversion may not be industrially prudent, although the IL may be recovered. Therefore, we suggest an optimum of 0.10 g IL/g Ca(OH)_2_ (10% IL), but in 30 min, because a conversion of 93.9% was obtained in this case, even though a 99.7% conversion was realized in 60 min.

The mechanism of the current SLGC system with Bmim[Br] is therefore similar to that of the SGC system with the solid ionic liquid previously reported [23,24]. Briefly, the Bmim[Br] absorbs water and CO_2_ to initiate the reaction by forming hydrated layers. These layers promote the dissolution of Ca(OH)_2_ to produce Ca^2+^ species and the conversion of CO_2_ to CO_3_^2−^ species. Because of the relatively high solubility of CO_2_ in Bmim[Br], there is enough CO_3_^2−^ avoiding the formation of the usual protective and passivation CaCO_3_ layer, and this led to the formation of stoichiometric rhombohedral calcite particles.

### 2.7. Brunner-Emmet-Teller (BET) Surface Area and Thermogravimetric Analysis (TGA)

BET analysis was done to study the specific surface area and pore structure of the sample synthesized with the IL at 5.0 MPa and 30 °C in 5 min, since this gave almost complete conversion (99.1%). A surface area of 13.0 m^2^/g was obtained. This result is comparable with values reported for SLGCs with similar conditions [4,30], but better than values reported for some SGCs [27]. The highest surface area for a similar system, but with a rotating disc reactor unit at ambient conditions, was reported to be between 20.4 and 23.3 m^2^/g; however, the products were filtered through a 0.1-μm membrane [31].

**Figure 9:**
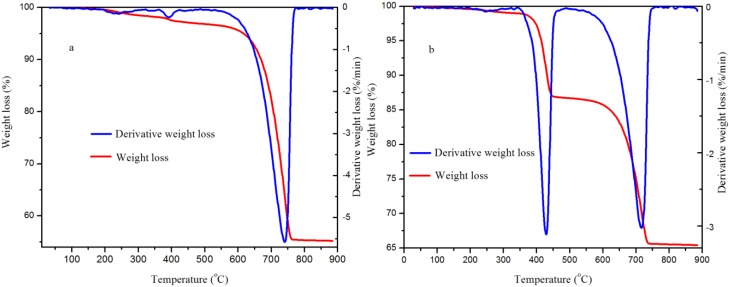
Thermogravimetric curves for the CaCO_3_ produced: (**a**) 5.0 MPa and (**b**) 0.1 MPa in 5 min at 30.0 °C.

The thermal stability of the samples produced at the lower and medium pressures was studied with a thermogravimetric analyzer. As shown in Figure 9, the total weight loss was 47.7% for the sample produced at 5.0 MPa, whilst a total weight loss of 34.6% was noticed for the sample produced at 0.1 MPa. The TGA results enabled the validation of the Rietveld whole pattern refinement of the XRD analysis (see Table 1). For this case, the weight loss due to the release of physically adsorbed water, the release of water from Ca(OH)_2_ and the release of CO_2_ from CaCO_3_ as a result of thermal decomposition were recorded, and the weight percent of CaCO_3_ in the sample was calculated with Equation (2).

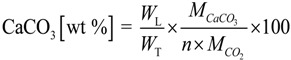
(2)
where *W*_T_ denotes the total weight of the sample (g), *W*_L_ denotes the weight loss (g), *n* denotes the number of moles of CO_2_ eliminated and *M* denotes molecular weight (g/mol).

**Table 1:** Validation of XRD Rietveld refinements by TGA.

Sample	Rietveld (wt %)	TGA (wt %)	Difference (wt %)
5.0 MPa	99.1	96.2	2.9
0.1 MPa	47.7	49.7	2.0

The endothermic peak at about 740.6 °C for the sample produced at 5.0 MPa led to about 96.2% (mass) CaCO_3_. Similarly, the endothermic peaks at about 428.6 °C attributed to decomposition of Ca(OH)_2_ and at about 716.6 °C assigned to the decomposition of CaCO_3_ for the sample produced at 0.1 MPa correspond to about 49.7% CaCO_3_. These percentage values are in agreement with the results from the Rietveld refinements for the XRD patterns (Table 1).

## 3. Experimental Section

### 3.1. Materials

Analytical grade Ca(OH)_2_ with 95% purity was purchased from Sinopharm Chemical and Reagents Co., Ltd, Shanghai, China. Carbon dioxide (99.99% purity) was sourced from Linde Gas, Xiamen Corporation Ltd., Xiamen, China, and 1-butyl-3-methylimidazolium bromide, Bmim[Br] with 99% purity was purchased from Shanghai Cheng Jie Chemical Co., Ltd., Shanghai, China.

### 3.2. Procedure

**Figure 10:**
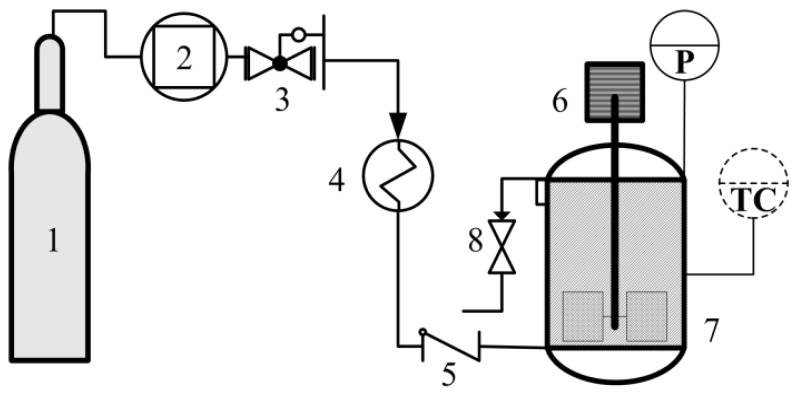
Illustration of the experimental setup: **1**, CO_2_ cylinder; **2**, compressor; **3**, back pressure regulator; **4**, preheater; **5**, check valve; **6**, mechanical stirrer; **7**, reactor; **8**, outlet valve; TC, temperature controller; P, pressure indicator.

The setup for the process is shown in Figure 10, consisting of a CO_2_ compressor (G447-400, Beijing HuiZhi M&E Facilities Co., Ltd., Beijing, China), a self-modified back pressure regulator (±0.1 MPa), a preheater, a check valve and a high pressure reactor (100 mL) with a maximum internal working pressure of 30 MPa, a temperature of 300 °C and outlet and inlet valves arranged, such that CO_2_ could be bubbled straight into the bulk of the slurry and discharged immediately after the reaction. The reactor is equipped with a mechanical stirrer (about 500 rpm), thermocouple, a pressure meter and a temperature controller (±0.1 °C).

Typically, an amount of distilled water (mL) was added to 5.0 g of Ca(OH)_2_ to produce a slurry. To this, Bmim[Br] of mass ratios of 0.5 g/5 g Ca(OH)_2_ was loaded into the reaction vessel and heated under stirring (500 rpm) until thermal equilibrium (°C). CO_2_ was fed into the vessel, brought to the desired pressure (MPa) and reacted for a set time (min). CO_2_ was deliberately released after the reaction; the product was collected, washed to recover the ionic liquid and dried in an oven (Jinhong, DZF-6020, Shanghai, China) at 100 °C for 12 h.

### 3.3. Characterization

The phase purity and quantity of the samples were analyzed with X-ray diffractometry (XRD), and the crystallite size was determined using Jade 5.0 from XRD patterns. Measurements of the XRD were carried out using X’Pert Pro (PANalytical B.V, Ea Almelo, The Netherlands) with a Cu anode operated at 40 kV, 30 mA and a wavelength (λ) of 1.540598 A. A continuous scan from 16° to 60° with a 2-theta step scan of 0.016711° and a step length of 10 s was employed. Calcite has a characteristic major peak at 29.41° corresponding to the {1 0 4} reflection plane, while Ca(OH)_2_ has a major peak at 34.11°, matching the {0 0 1} reflection plane, followed by the peak at 18.08° [24]. In our case, however, the Ca(OH)_2_ peak at 18.08° was the highest. Phase analysis and quantification was achieved by Rietveld whole pattern refinements using X’Pert HighScore plus (2.0) and the ICDD-PDF-2 database [24]. Specific surface area and pore size were determined by BET (Micrometric TRISTAR-3000, Micromeritics Instrument Corporation, Norcross, GA, USA), and isotherms were obtained by performing isothermal physical sorption with N_2_ gas at 77.3 K. SEM (S4800, Hitachi, Tokyo, Japan) was employed to study the morphology of the samples by dispersing samples ultrasonically in absolute ethanol for 30 min and depositing on a silicon wafer. Thermal stability was studied by thermogravimetric analysis (NETZSCH TG 209 F1, Selb, Germany) under N_2_ from 30 to 900 °C with a heating rate of 10 °C/min.

## 4. Conclusions

We investigated the effect of several variables on an SLG carbonation system using Ca(OH)_2_ slurry with an imidazolium-based IL, Bmim[Br]. Based on the results, we conclude that: (1) the use of the IL positively influenced the carbonation reaction at relatively low pressure (0.1 MPa), such that with 1 g IL/1 g Ca(OH)_2_, a conversion higher than 94.4% was achieved within 5 min; (2) the optimum condition (0.10 g IL/1 g Ca(OH)_2_ in 30 min) at the lower pressure gave a conversion of 93.9%; (3) medium pressure (5.0 MPa) led to almost complete conversion (99.6%) in 5 min; (4) 5.0 mL of water or more was necessary for the rapid conversion of 5.0 g Ca(OH)_2_, yet the removal of water from the system gave very low conversion; (5) the samples exhibited rhombohedral calcite structures with a high specific surface area; and (6) the amount of CaCO_3_ obtained from the Rietveld refinement of XRD patterns was very close to that obtained from TG analysis.
